# *Lacticaseibacillus paracasei* subsp. *paracasei* 2LB: Identification of Genes to Assess the Safety and Probiotic Potential of the Strain

**DOI:** 10.3390/foods14193449

**Published:** 2025-10-09

**Authors:** Gulyaim Abitayeva, Diana Kurmangali, Temirlan Baikonys, Zhandarbek Bekshin

**Affiliations:** Republican Collection of Microorganisms LLP, Astana 010000, Kazakhstan; d.qurmangali@gmail.com (D.K.); temirlanbaikonys1@gmail.com (T.B.); zmbekshin@gmail.com (Z.B.)

**Keywords:** *Lactocaseibacillus paracasei* subsp. *paracasei*, whole genome sequencing, carbohydrate-active enzymes, safety and stability assessment, probiotic related multiple genes, probiotic potential, bacteriocins

## Abstract

In this study, we conducted a whole-genome analysis of the *Lacticaseibacillus paracasei* subsp. *paracasei* 2LB isolated from Kazakh traditional fermented milk (koumiss) to identify genes associated with the safety and probiotic potential of the strain. A comparative genomic analysis of the core and pan-genome of *L. paracasei* 2LB was performed. Functional annotation revealed the presence of genes putatively involved in metabolism, genetic information processing, and cellular processes. In terms of safety parameters, the stability of its genetic material, the absence of the ability to synthesize virulence factors, and genes responsible for antibiotic resistance were characterized. Also, in vitro studies of the *L. paracasei* 2LB strain showed resistance to stress factors and antimicrobial activity, and the presence of coding sequences encoding adhesion factors, bacteriocins, bile salts, pH, cold and heat shock, and osmotic stress was observed through genomic analysis. These results indicate that the *L. paracasei* 2LB strain is a potential probiotic candidate and demonstrate that whole-genome analysis is a useful method for assessing the quality and safety of probiotics.

## 1. Introduction

Using probiotics can have a beneficial effect on the balance of gastrointestinal microbiota and the immune system’s ability to function properly [1,2,3,4,5,6]. Among probiotic microorganisms, species that were classified as *Lactobacillus* according to the previous taxonomy prior to 2020 are commonly used [7]. Most of these bacteria have “Generally Recognised as Safe” (GRAS) status [8,9] and a Qualified Presumption of Safety (QPS) [10]. In fact, it has been reported that some lactobacilli can be unsafe [11,12]. *Lacticaseibacillus paracasei* subsp. *paracasei* (*L. paracasei*) is one of the most prominent representatives of Gram-positive lactic acid bacteria, known for its beneficial properties and applications in various areas of life [13,14,15]. *L. paracasei* is most widely used as a probiotic in various foods, mainly in meat and fermented dairy products [9]. Their phenotypic characteristics, namely obligate/facultative and homo/heterofermentative abilities, play a decisive role in the fermentation of raw milk and in the production of fermented milk products [7], namely fermented milk, cheese, yogurt, and probiotics [16]. *L. paracasei* is known for its special role in cheese production, which is associated with unusual taste, aroma, and improved product quality.

Studies of the strain *L. paracasei* have shown that this type of bacteria improves the condition of the gastrointestinal tract, reducing the duration of common gastrointestinal infections and decreasing the frequency of diarrhea caused by antibiotic use [14,17,18], by strengthening the epithelial barrier and reducing the amount of harmful substances and pathogens, as well as producing beneficial substances such as short-chain fatty acids and carboxylic acids [5].

Currently, researchers are also focusing on whole-genome analyses of probiotic microorganisms, which identify the presence of genes associated with antimicrobial resistance, virulence factors, and toxic metabolites that pose a potential health risk. New data based on whole-genome sequencing (WGS) analysis complements knowledge in the field of probiotic safety research [19]. As of the current date, there is data available on the complete genome sequence of *L. paracasei* and its genetic analysis in silico, as well as research on its potential probiotic properties using bioinformatic tools [20,21]. This approach provides a deep understanding of their genetic information, evolutionary diversity, and metabolic characteristics at the gene level and is important for further understanding the molecular mechanisms associated with probiotic properties [14].

The strain *L. paracasei* 2LB was isolated as part of our research from koumiss. Koumiss, produced by lactic acid and alcoholic fermentation of mare’s milk, is part of Kazakhstan’s cultural and biological heritage. It is highly regarded for its complex microbiological composition and healing properties, particularly in Central Asian cuisine. Since the consumption of traditional fermented milk products plays a significant role in providing important nutrients necessary for health, it is important to obtain probiotic strains with satisfactory technological properties from local milk products that are safe and suitable for use in the food and pharmaceutical industries. The promising microorganisms that have been isolated from it not only expand our knowledge of regional biodiversity but also lay the scientific foundations for developing functional foods and biotechnological solutions. An in-depth characterization of the properties of *L. paracasei* is of theoretical interest and has practical significance in the targeted design of new effective probiotic products, taking into account their use by individuals and groups of people depending on their region, gender, age, and physiological condition.

The aim of this study is to investigate the strain *L. paracasei* 2LB using WGS and genome analysis to identify its probiotic potential and assess its safety and further application in various types of food products or biomedicine.

## 2. Materials and Methods

### 2.1. L. paracasei 2LB Isolation and Cultivation Conditions

*L. paracasei* 2LB (B-RKM 0545) was isolated from the Kazakh homemade traditional drink koumiss, Akmola region (Kazakhstan). The strain was identified using standard tests including morphological, biochemical, and physiological methods. The analysis also included 16s rRNA sequencing and further comparison with strains in BLAST (NCBI, Bethesda, MD, USA). Culture seeding was performed in De Man–Rogosa–Sharpe broth (Condalab, Madrid, Spain) at pH 6.5 and incubated at 37 °C for 24 h. The initial cultures in MRS broth were mixed with a cryo medium, except for 20% glycerol (PanReac AppliChem, Darmstadt, Germany) and 10% sucrose, and stored at −80 °C (TM Media, Delhi, India).

### 2.2. Whole-Genome Sequencing and De Novo Assembly

Full genome sequencing of the *L. paracasei* 2LB strain was performed on the Illumina MiSeq Reagent v3 600-cycle platform (Illumina, San Diego, CA, USA). The FastQC v0.12.1 “https://github.com/s-andrews/FastQC (accessed on 20 August 2024)” program was used to control the quality of reads. SeqTK v1.4 “https://github.com/lh3/seqtk (accessed on 20 August 2024)” and Sickle v1.33 “https://github.com/najoshi/sickle (accessed on 20 August 2024)” programs were used to trim the reads and SPAdes v3.15.5 “https://github.com/ablab/spades (accessed on 20 August 2024)” was used for de novo genome assembly. The quality of assembly was assessed by the QUAST v5.2.0 program “https://github.com/ablab/quast (accessed on 20 August 2024)”. For species identification, we used BLASTn comparison of orthologous groups of assembled contigs with the database “refseq_prok_rep_genomes” (Prokaryotic representative genomes from NCBI). Implemented in Python v3.12, we used the OrthoANI v0.7.0 program algorithm to compare large genomic sequences by determining “average nucleotide identity” [22]. The Prokka v1.14.5 program performed annotation of genome assemblies [23]. The whole-genome sequence data for *L. paracasei* 2LB were submitted to GenBank under access number JBQPXO000000000.

### 2.3. Phylogenomic Analysis of the Core- and Pan-Genome and Functional Annotation

A total of 93 strains of *L. paracasei* were used, obtained from the NCBI GenBank databases (query from August 2024). The original genomes of *L. paracasei* were annotated using Prokka v1.14.5 (GFF format), and further mistake correction, clustering, and pan- and core genome identification were performed using Panaroo v1.5.1. The ‘–clean-mode strict’ and ‘–identity 0.98’ options were used to combine genes with high similarity and minimal false clustering. The RapidNJ “https://github.com/somme89/rapidNJ (accessed on 27 November 2024)” method based on the Neighbor Joining algorithm with 1000 bootstraps was used to construct the core- and pan-genome tree.

Using genomic sequence databases from EggNOG v5.0, orthologous groups (COGs) were categorized based on their functions [24]. Proteins were categorized into KEGG Orthology (KO) groups and KEGG maps were produced using BlastKOALA (version 2.2) [25]. CAZyme annotation was performed using the dbCAN3 metaserver [26]. Bacteriocins were predicted using the BAGEL4 program [27].

To identify annotated genes associated with probiotic functions, a keyword search was performed in annotation files in eggNOG-mapper v 2.1.12 [28], functional subsystems on the RAST platform [29], and comparison with reference protein sequences via BLASTp [30].

### 2.4. In Silico Safety and Stability Assessment

To evaluate a potential probiotic, the stability and safety of the genetic material was considered, which is due to the absence of virulence factors, plasmids, and mobile elements and genes responsible for antibiotic resistance. Virulence factors were determined using VirulenceFinder v2.0 program [31]. We searched for mobile genetic elements using the oriTfinder2 program [32].

Antibiotic resistance genes were searched in RGI 6.0.5 web-portal (CARD 4.0.1) “https://card.mcmaster.ca/analyze/rgi (accessed on 28 July 2025)”, ResFinder v4.0 [33]. Pathogenic genes were searched using the PHI-base v4.17 “http://www.phi-base.org/index.jsp (accessed on 20 June 2025)” and PathogenFinder 1.1 [34]. The presence of plasmids was detected using PlasmidFinder 2.1 [35]. PHASTER and WtP programs were used to determine prophage sequences [36]. CRISPR-Cas identification and characterization were performed using CRISPRCasFinder [37].

### 2.5. Data Visualization

Bar charts were constructed using GraphPad Prism v9.5.1. (GraphPad Software, Boston, MA, USA) “https://www.graphpad.com/ (accessed on 9 March 2024)”. Genome visualization was performed using the Proksee v1.2.0 program [38].

## 3. Results and Discussion

In this study, we investigate the *L. paracasei* 2LB strain for probiotic value. To use the *L. paracasei* 2LB strain as a probiotic base, it must meet the criteria for qualifying microorganisms as “probiotics” in foods and supplements [39] and comply with the QPS, which requires that the assessment of probiotics includes identification, subject of assessment, safety concerns, and antimicrobial resistance [40].

### 3.1. Genome Features of the L. paracasei 2LB

**Figure 1:**
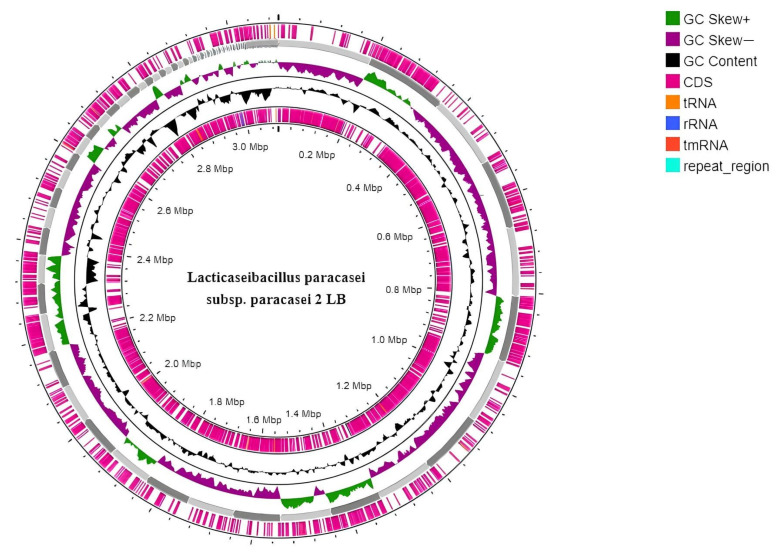
Circular map of the *L. paracasei* 2LB genome, created using Proksee v1.2.0 software. From the outer circle to the inner circle, the following genomic features are presented: CDS (pink), rRNA genes (blue), tRNA genes (orange), GC content (black), GC Skew+ (dark green), and GC Skew− (purple).

The genomic features of *L. paracasei* 2LB were studied using WGS, followed by de novo assembly and annotation of the resulting genome, which was visualized using the Proksee program (Figure 1).

The genome of *L. paracasei* 2LB consists of a single linear chromosome. The characteristics of this genome were determined using assembly and structural annotation data. The analysis identified key genetic features, including gene organization and functional elements (Table 1).

As a consequence, in assembling the *L. paracasei* 2LB genome, the total length is 3,066,038 bp, GC 46.25%, and consists of 219 contigs. A total of 2932 coding sequences, 59 tRNAs, six rRNAs, and two CRISPR arrays were identified in the genome. The N50 value for *L. paracasei* 2LB was 105,408 bp, and the sizes of the longest and shortest contigs were 195,833 bp and 129 bp, respectively.

**Figure 2:**
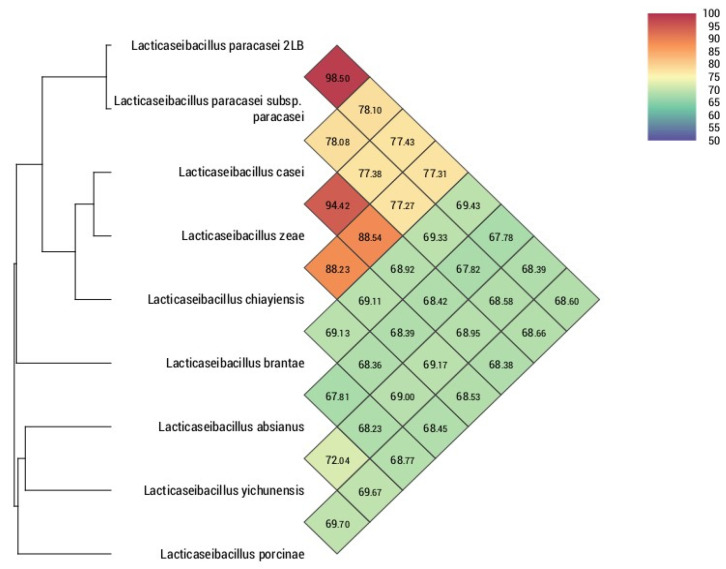
Results of orthologous average nucleotide identity between *L. paracasei* 2LB and other *Lacticaseibacillus* species.

To confirm the uniqueness of the strain, the average nucleotide identity (ANI) was used as a metric, and a comprehensive phylogenetic analysis was used to confirm its belonging to the species *L. paracasei* (Figure 2).

Figure 2 of the OrthoANI heat map shows that the genome under study was found to be consistent with the reference strain *L. paracasei* 2LB, with an ANI value of 98.50%, significantly exceeding the generally accepted ANI threshold value (95–96%) commonly used to distinguish prokaryotic species [41].

### 3.2. Phylogenomic Analysis of the Core- and Pan-Genome

**Figure 3:**
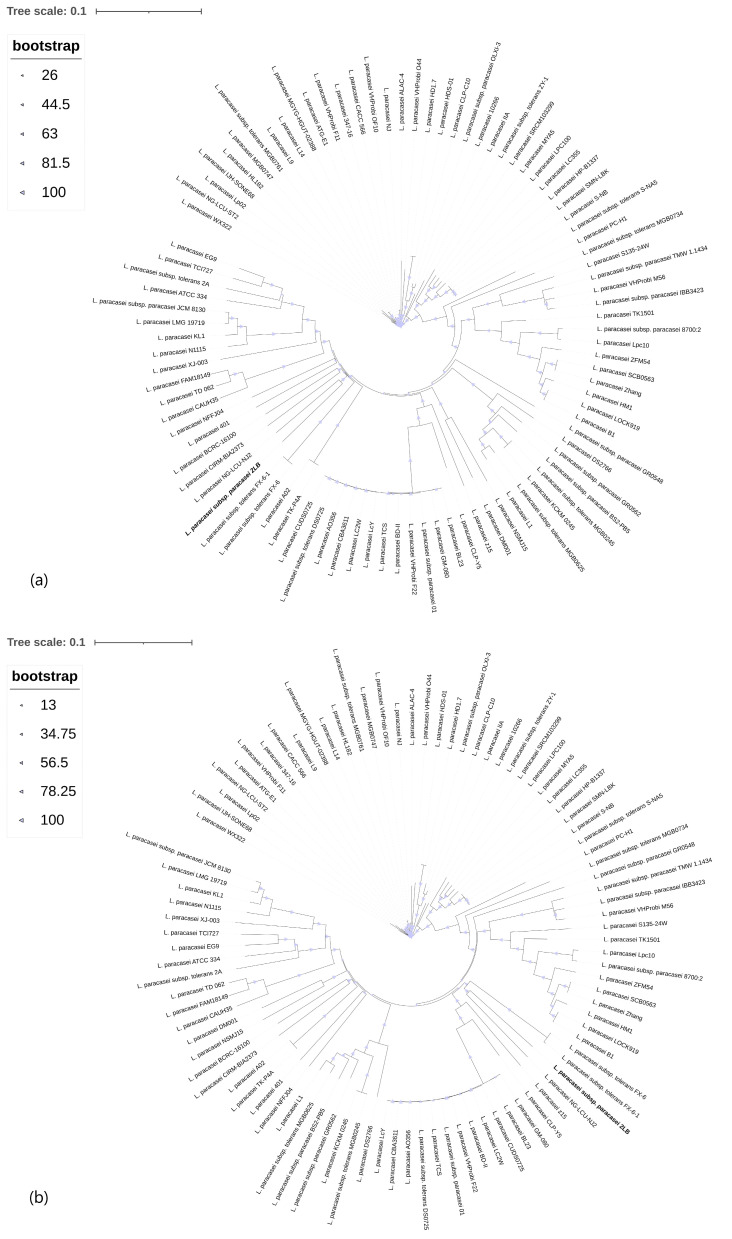
Maximum-likelihood phylogenetic tree for 94 complete *Lacticaseibacillus paracasei*, including strain *L. paracasei* 2LB, constructed using 1000 bootstrap repetitions with the ITOL program: (**a**) core-genomes; (**b**) pan-genomes.

For the analysis of core- and pan-genomes, a comparative genomic analysis of *L. paracasei* 2LB was performed with 93 full-genome strains of *L. paracasei* taken from the NCBI database (Table S1) in order to identify genomic differences at the subspecies level, with particular attention paid to unique genes responsible for the manifestation of adaptive and probiotic characteristics. A maximum-likelihood tree was constructed based on wgSNP (whole genome SNPs) using the kSNP program with an optimal Kmer value of 19 [42]. The tree was rooted by adding *L. rhamnosus* (NZ_CP040780.1) to the analysis. The results of the phylogenetic analysis based on core-genomes and pan-genomes are presented in Figure 3.

The study of the pan-genome, consisting of core- and accessory genomes, and also the core-genome, containing conserved orthologous genes, is closely related to species relationships and gene exchange, and this reflects species stability [43]. Based on the core- and pan-genome, a phylogenetic tree analysis was performed, which indicates significant genetic homology between *L. paracasei* 2LB and the reference *L. paracasei* NG-LCU-NJ2 (GCF_027886355.1) (Table S1). The pan-genome homology results showed 99%, indicating minor changes in the periphery of the *L. paracasei* 2LB genome. At the same time, the core-genome similarity indicators were 100%, indicating close phylogenetic relationship and conservative evolution.

### 3.3. Functional Annotation

**Figure 4:**
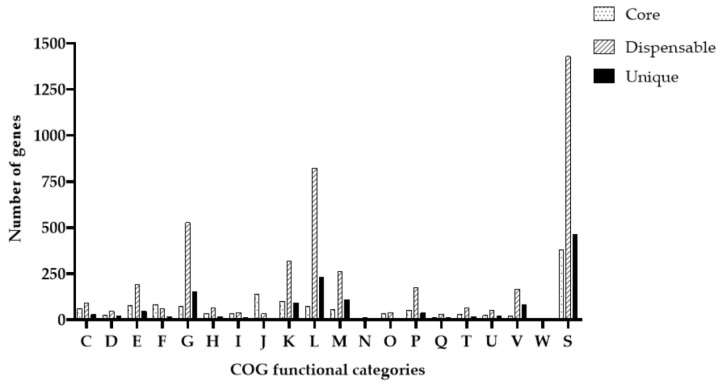
Functional annotation of proteins from the strain *L. paracasei* 2LB according to the COG database. COG functional categories: C—Energy production and conversion; D—Cell cycle control, cell division, chromosome partitioning; E—Amino acid transport and metabolism; F—Nucleotide transport and metabolism; G—Carbohydrate transport and metabolism; H—Coenzyme transport and metabolism; I—Lipid transport and metabolism; J—Translation, ribosomal structure, and biogenesis; K—Transcription; L—Replication, recombination, and repair; M—Cell wall/membrane/envelope biogenesis; N—Cell motility; O—Post-translational modification, protein turnover, chaperones; P—Inorganic ion transport and metabolism; Q—Secondary metabolites biosynthesis, transport and catabolism; S—Function unknown; T—Signal transduction mechanisms; U—Intracellular trafficking, secretion, and vesicular transport; V—Defense mechanisms; W—Extracellular structures.

To determine the functions performed, we extracted sequences of core genes for comparison with the COG database (Figure 4). Clustering and identification of pan- and core-genomes were performed using the Panaroo v1.5.1 program, which classified genes into three categories: core, dispensable, and unique proteins. In the *L. paracasei* 2LB strain, 1329 core genes, 1370 dispensable genes, and 30 unique genes were identified.

To study functional annotation, protein sequences were divided into 20 COG categories. The results showed that dispensable genes (50.20%) were the most represented of the three categories, while the percentage of unique genes and core genes in the genome was 1.10% and 48.70%, respectively.

The most common category among COGs is “Function unknown” genes (S, 29.20%). The next category of genes is “Carbohydrate transport and metabolism” (G, 9.53%), followed by “Transcription” (K, 8.10%) and “Amino acid transport and metabolism” (E, 7.15%).

*L. paracasei* 2LB has 30 unique genes, including 15 proteins with known functions and 15 “Function unknown”. Among the 15 functionally known unique genes present in the 2LB genome, 4 genes are classified as “M; Cell wall/membrane/envelope biogenesis”. These genes contain several families of glycosyl transferase and peptidoglycan-binding protein, which support the strength and integrity of bacterial cells by forming glycosidic bonds and assembling peptidoglycan and other polysaccharide structures. In the “L; Replication, Recombination, and Repair” category, three genes were identified whose activity is directed toward DNA manipulation. Specifically, HNH nucleases cleave the complementary strand [44], ATP-binding protein rearranges it [45], and the helix-turn-helix domain specifically binds to it. This ensures genetic flexibility, stress resistance, regulation of gene expression, and the integration or protection of foreign genetic components. Two genes classified as “V; Defense mechanisms” have a protective function. They are capable of recognizing foreign DNA and then directing an enzymatic complex to degrade it. In addition, two genes belonging to the category “K; Transcription” are involved in the process of transcription regulation, acting as both repressors and activators. In addition to the genes already mentioned, others were classified into one of the following functional groups: “D; cell cycle control, cell division, and chromosome distribution,” “G; carbohydrate transport and metabolism,” “I; lipid transport and metabolism,” and “P; inorganic ion transport and metabolism.”

**Figure 5:**
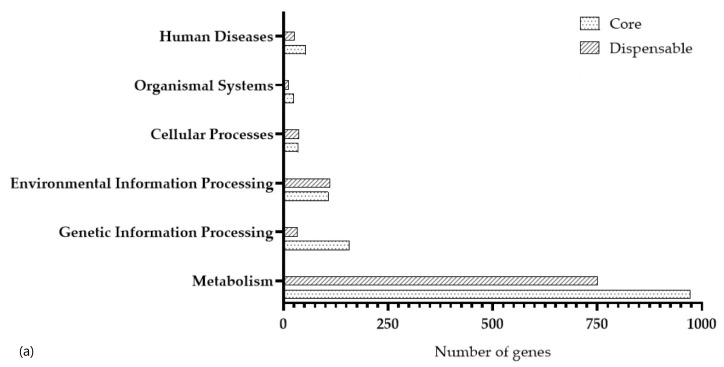

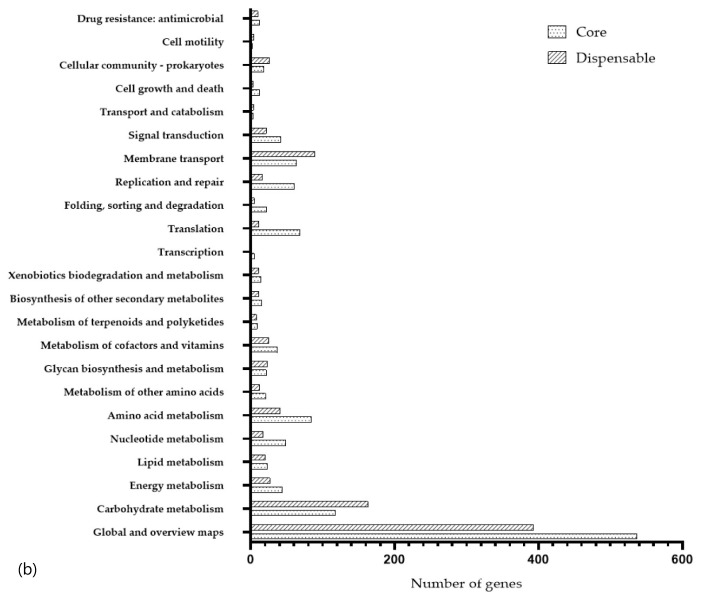
Functional annotation of genes from the strain *L. paracasei* 2LB according to the KEGG database: (**a**) main categories; (**b**) subcategories.

Figure 5 demonstrates 2323 functionally annotated genes identified using the KEGG database and classified into core, dispensable, and unique groups, as well as distributed according to their functional categories.

Figure 5 clearly demonstrates the differences in the number of genes belonging to each group and their contribution to various biological processes and functions. As shown in Figure 5a, genes are divided into six major categories, with “Metabolism” (n = 1611), followed by “Environmental Information Processing” (n = 199), “Genetic Information Processing” (n = 178), “Human Diseases” (n = 78), “Cellular Processes” (n = 66), and “Organismal Systems” (n = 33).

The number of genes classified ranged from large to small: the largest number belonged to the “Metabolism” category, a moderate number to “Transcription, Replication, Recombination, Repair and Membrane Transport”, and a smaller number to “Organism Systems”. Based on these data, assigning KEGG to functional categories resulted in the classification of the predicted genes into 40 subcategories (Figure 5b). Within the “Metabolism” category, the largest subcategory was “Global and Overview Maps” (n = 874 in total), 537 of which belonged to core genes. “Global and Overview Maps” is an integrated map showing common metabolic networks and cross-links between major pathways. The next largest subcategories were the *L. paracasei* 2LB “Carbohydrate metabolism” genes (n = 253), followed by the “Membrane transport” genes (n = 874) and the “Amino acid metabolism” genes (n = 115).

### 3.4. CAZymes Carbohydrate-Active Enzymes Analysis

The carbohydrate-active enzymes (CAZy) database mainly contains glycoside hydrolases (GH), glycosyltransferases (GT), polysaccharide lyases (PL), carbohydrate esterases (CE), and auxiliary activities (AA). CAZy genes in genome sequences were predicted using the dbCAN3 metaserver with the HMMER tool (Table 2).

As a result of the analysis of the *L. paracasei* 2LB genome, 85 carbohydrate-active enzymes (CAZymes) (Table S2) were identified, among which the main predicted categories are glycoside hydrolases (GH) and glycosyltransferases (GT), which account for 47.06% and 38.82% of the total number of enzymes represented in the genome, respectively. Next are carbohydrate esterases (CE, 5.88%), auxiliary activities (AA, 3.53%), and carbohydrate-binding modules (CBM), and polysaccharide lyases (PL) showed the same percentage of genes, equal to 2.35%. The set of 40 GH (glycoside hydrolase) genes was grouped into 18 different families, among which the GH13 family (n = 8) dominates, participating in the breakdown of starch, glycogen, and other polysaccharides by catalyzing the hydrolysis of α-1,4- and α-1,6-glycosidic bonds [13].

The second most common is GH25 (n = 6), which is associated with the lysis of bacterial cell walls and antimicrobial activity. Thus, the GH genes in the genome profile indicate the pronounced ability of the *L. paracasei* 2LB strain to utilize carbohydrates and compete with microorganisms.

In the studies of Ghosh S. et al., it was observed that cellulose synthase (GT2), a key enzyme in the synthesis of polysaccharides, which provides protection from external influences due to cell attachment and biofilm formation, also predominates among 75 strains of *L. paracasei* [13]. GT4 is also involved in the transfer of carbohydrates during the biosynthesis of oligosaccharides and lipopolysaccharides of the cell wall and capsules in microorganisms. Due to the presence of the CAZymes genes—GH and GT—*L. paracasei* strains are able to metabolize various carbohydrates and inhabit different ecological niches [46,47], thereby demonstrating adaptive characteristics.

### 3.5. Genomic Analysis of Bacteriocins

**Figure 6:**
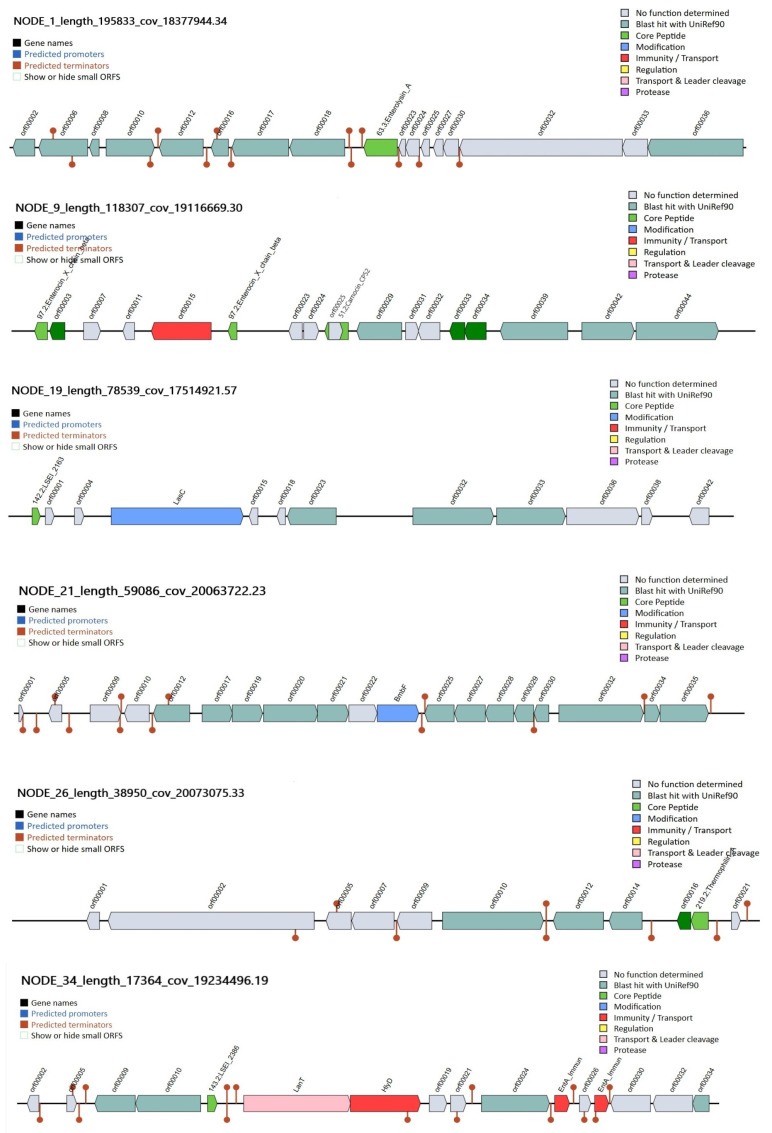
Visualization of annotated clusters of genes encoding antimicrobial bacteriocins in the genome of *L. paracasei* 2LB based on data from the BAGEL4 database.

Using the BAGEL4 web server, antimicrobial bacteriocin clusters (Sactipeptides, Thermophilin_A (ThmA), LSEI_2163, LSEI_2386, Carnocin_CP52, Enterolysin_A) were found in the genome of the *L. paracasei* 2LB strain (Figure 6).

Bacteriocins perform an antimicrobial function and affect the growth of microorganisms, based on a mechanism that consists of membrane destruction in the *L. paracasei* 2LB strain; six contigs NODE_34, NODE_19, NODE_21, NODE_9, NODE_26, and NODE_1 characterized by the presence of bacteriocins were identified (Figure 6). Contigs NODE_34_length_17364_cov_19234496.19 (13,978 bp, 16 ORF) and NODE_19_length_78539_cov_17514921.57 (10,459 bp, 12 ORF) encode Class IIb bacteriocins LSEI_2386 and LSEI_2163, respectively. Kuo YC et al., in a study of bacteriocins LSEI_2163 and LSEI_2386, found that they showed antimicrobial activity against some species of *Lactobacillus* and *Listeria* [48].

Contig NODE_21_length_59086_cov_20063722.23 (20,000 bp, 9 ORF) produces Sactipeptides. Sactipeptides are known to improve stability and exhibit selective activity against antibiotic-resistant bacteria [49,50,51].

The content of 18 ORF potentially coding regions was detected in the contig NODE_9_length_118307_cov_19116669.30. Enterocin_X_chain_beta was detected in ORF00002 and ORF00017 with a match level of 50.98% (E-value = 1.62 × 10^−13^) and 68.00% (E-value = 1.62 × 10^−10^), respectively. Genomic analysis revealed clusters ORF00003, ORF00033, and ORF0003 encoding Class IIb bacteriocin, and a segment of ORFBLAST_1 encoding Carnocin_CP52 (333 bp). Carnocin_CP52 has high antimicrobial activity against Gram-positive pathogens [52].

Contig NODE_26_length_38950_cov_20073075.33 consists of 11,011 bp and includes 11 ORF segments, where ORF00016 is identified as Class IIb bacteriocin and ORF00019 as Thermophilin_A (Type-A lantibiotic; Evaluate = 3.71 × 10^−10^ match = 41.667%). Bacteriocin Thermophilin_A has a wide range of antimicrobial activity, inhibiting the growth of closely related strains and competing species [53], thereby providing an advantage in the nutritional niche [54].

Contig NODE_1_length_195833_cov_18377944.34 encodes Enterolysin_A with a 50.617% match (Evalue = 2.42 × 10^−22^). The presence of the Enterolysin_A gene is common among probiotic strains of bacteria [55,56], which inhibits the growth of enterococci, pediococci, lactococci, and lactobacilli [57].

The presence of similar bacteriocin gene clusters in the *L. casei/paracasei* genome was also reported by Surachat K et al. and Kandasamy et al. in the analysis of potential probiotic strains [46,58]. In silico analysis predicts the ability of strain 2LB to produce bacteriocins of various classes, which may exhibit effective antagonistic activity against pathogens. In the study of antimicrobial activity, strain 2LB showed the presence of an inhibition zone against several pathogens such as *Candida albicans*, *Escherichia coli*, *Salmonella typhimurium*, and *Staphylococcus aureus* (Table S4). However, additional in vitro studies confirming the production of bacteriocins are needed.

### 3.6. Probiotic Related Multiple Genes Presented of the L. paracasei 2LB Strain

Under stress conditions, a number of genes are induced (Table 3) which are involved in physiological and molecular responses to improve growth, adaptation, and survival, thereby mitigating the effects of stress [9].

Bacteria possess complex adaptation systems to various stressful conditions, including heat, cold, osmotic pressure, and bile salts, during which many specific proteins are expressed (Table 3). Genes encoding stress-associated proteins have been identified, indicating the probiotic potential of the *L. paracasei* 2LB strain. However, only some of these functions have been confirmed in separate in vitro experiments. (Figure S1 and Table S4) [110].

In the strain *L. paracasei* 2LB, genes were identified encoding cold shock proteins (CspA, CspC, CspG and CspR), which are activated by a change in temperature to the stress response [66], and heat shock proteins (the DnaKJ and GroESL complex, LepA, Hsp1, Hsp3, etc.), which participate in RNA binding, preventing the formation of secondary structures, maintaining the integrity of cellular structures, as well as in post-transcriptional regulation of a number of genes [56]. Proteases ClpC, HtrA, and FtsH were found, which are involved in cellular protein quality control, namely in the reactions of degradation, turnover, and destruction of misfolded, damaged proteins [80], and the absence of these proteases in *Lactobacillus* cells impairs growth under conditions of constant heat or hyperosmotic stress [111]. YabA, acting as a concentrator protein, regulates the initiation of DNA replication by interacting with other partner proteins [71,72]. The presence of genes encoding cold shock and heat shock proteins in the *L. paracasei* 2LB strain may play an important role in protecting the cell from extreme temperatures [112]. This property is of significant technological importance in the preparation of fermented milk products for stages such as pasteurization, as well as in the application of various temperature regimes in food production [113].

During the study of the genome of *L. paracasei* 2LB, genes encoding proteins of the superfamily of membrane channels “ABC transporters” (OpuAA, OpuAC, OpuCA, OpuCB, OpuCC, OpuAB, OpuCD) were identified, which carry out active transport of osmolytes (glycine betaine, L-carnitine, choline) and are linked to ATP hydrolysis [85]. The presence of a mechanosensitive MscL channel promotes the rapid release of osmolytes and cell stabilization [84]. The increased osmotic stability of lactic acid bacteria is associated with the activity of the proX and proV genes, which are responsible for the synthesis of ABC permeases, the L-proline-glycine-betaine transporter [87]. A study of osmotic stress resistance of strain 2LB showed the ability to grow in the presence of 2–6.5% NaCl (Figure S1), and the discovered genes responsible for the absorption, transport, and accumulation of osmoprotectors make strain *L. paracasei* 2LB a worthy candidate in the food industry [46,114].

The operon atpBEFHAGDC, consisting of eight genes (atpB, atpE, atpF, atpH, atpA, atpG, atpD, and atpC) [106], was found in the studied strain 2LB. It encodes the F0F1ATPase complex [115], which is involved in the generation of proton motive force (PMF) [113] and helps the cell cope with the increased acidity of the internal environment [105]. The 2LB strain was well-tolerated in a simulated gastrointestinal environment and grew well at pH = 3.5 (Figure S1). Based on the data obtained, it can be assumed that the resistance of *L. paracasei* 2LB to low pH conditions is due to the presence of these factors, which is consistent with the conclusions made by Kandasamy S. and Li J. [46,116].

The bile salts resistance genes (mleP and mleS MurE, genes encoding choloylglycine hydrolase) were identified in the genome of *L. paracasei* 2LB. Along with this, an in vitro study of the resistance of *L. paracasei* 2LB to bile salts showed a good growth of 0.3–1.0% of bile salts (Table S4). The manifestation of protective mechanisms can be caused by the synthesis of enzymes of the choloylglycine hydrolase family, which deconjugate acids and reduce their toxicity [90,91]. According to Ma X. et al., activation of genes (mleP and mleS) involved in the MLE pathway (conversion of dicarboxylic acid L-malate to L-lactate and carbon dioxide) supports membrane homeostasis, which increases the strain’s resistance to bile stress [90]. In addition, MurE participates in the modification of the cell wall and metabolic pathways, which further enhances the resistance of the bacterium [92]. Thus, the combination of these mechanisms suggests that *L. paracasei* 2LB is highly adapted to the adverse conditions of the gastrointestinal tract.

The 2LB strain had high rates of autoaggregation and coaggregation to pathogens (Table S4). The detection of adhesion factors in the 2LB genome may determine the complex adhesive properties of the strain associated with the expression of polysaccharides (epsG and epsB) [93], specific surface antigens (p40 and p75) [117], and adhesive proteins (FbpA [96], PdhB [102], MapA [118], и InlJ [98]). These factors determine the potential of *L. paracasei* 2LB for biofilm formation, increased survival, and play a crucial role in protecting against inflammation and maintaining intestinal epithelial homeostasis [100].

Thus, the assessment of the probiotic potential of the *L. paracasei* 2LB strain using sequencing and genomic analysis in combination with in vitro studies, as well as future comparisons of the 2LB strain with other food-related probiotic bacteria (*Lactiplantibacillus plantarum*, *Bifidobacterium* spp., etc.), will help expand the 2LB strain into a broader range of probiotic applications. This is important for understanding the functional mechanisms and will thus impact fundamental and applied probiotic research.

### 3.7. In Silico Safety and Stability Assessment

A comprehensive safety assessment of probiotic strains is mandatory according to FAO/WHO recommendations, the European Food Safety Authority (EFSA, 2024), and the GRAS principles established by the Food and Drug Administration (FDA, USA). This assessment should include both molecular approaches, such as whole-genome sequencing (WGS), and confirmatory phenotypic studies [119,120].

In this study, the safety and stability profile of the *L. paracasei* 2LB strain was investigated in silico. Three complete (intact) prophage sequences and one incomplete sequence (due to the small size of the contig) were identified in the genome of the *L. paracasei* 2LB strain using the PHAge Search Tool Enhanced Release (PHASTER) and WtP in contigs “NODE_1”, “NODE_5”, “NODE_8”, and “NODE_29”, with sizes of 32.8 bp, 43.5 bp, 41.8 bp and 26.3 bp, respectively [17].

Using the PlasmidFinder 2.1 program, the absence of plasmid contigs was identified based on the presence of typical plasmid genes, such as replication genes and mobilizable plasmids, as well as based on similarity to previously published plasmid sequences in the NCBI database “https://www.ncbi.nlm.nih.gov/ (accessed on 20 February 2025)”. Studying the presence of plasmids [121,122], mobilizable elements (IMEs) are the first step in studying genome stability [109], and identifying the presence or absence of horizontal gene transfer [123]. Analysis using oriTfinder2 revealed the absence of mobile genetic elements including the oriT region [124,125], relaxase genes [126], type IV coupling protein (T4CP) [127], and type IV secretion system (T4SS) gene clusters [128]. As a result of using the VirulenceFinder v2.0 program, no virulence genes were found by performing a BLAST search on the collected genomic data (Table S3). This absence is a favorable indicator, as it reduces the risk of horizontal gene transfer of virulence and antimicrobial resistance [109,129], which is consistent with the safety criteria of probiotic strains.

The search of pathogenic genes in the genome of *L. paracasei* 2LB using the PathogenFinder 1.1 program showed a zero score for Pathogenic Families, a score of 492 for Non-Pathogenic Families, and a low probability for being a human pathogen with a value of 0.188 (Table S3). Thus, the prediction of human pathogenicity of the 2LB strain showed non-pathogenicity and safety [34].

Along with this, analysis of pathogenic genes using the PHI-base v4.17 program identified two pathogenic genes, WalR and CspR, with identities of 83.40% and 89.39%, respectively (pident > 80, qcovhsp > 90) (Table 4).

The WalR and CspR genes are multifunctional, providing essential regulatory and adaptive processes. WalR is an essential gene that regulates gene expression in the metabolism of the bacterial cell wall [130,131]. In the genomic safety analysis of the *Lactobacillus mucosae* strain, the pathogenic gene WalR was similarly detected; however, the strain was determined to be safe based on a comprehensive assessment of antibiotic resistance factors, virulence factors, and pathogenicity [132]. The CspR gene plays a role in long-term cell survival by regulating the expression of cold shock proteins in response to bacterial stress [65]. As these genes are likely to perform regulatory and auxiliary functions in non-pathogenic strains, their presence in 2LB strain’s genome may not directly indicate potential pathogenicity. Their possible pathogenic effect is determined not simply by their presence, but by possible expression levels and interactions with other factors, which should be studied in more detail.

The ResFinder v4.0 program did not detect any signs of phenotypic resistance to antibiotics in the genome of *L. paracasei* 2LB. Analysis using the RGI 6.0.5 (CARD 4.0.1) web portal programs revealed a region encoding the resistant protein qacJ with a percentage of identity of 38.24%, which provides unreliable information (Table S3).

The results obtained indicate the safety of strain 2LB, which meets the EFSA global safety criteria due to the absence of antibiotic resistance genes and virulence factors [119], and GRAS (FDA) status, which means it can be used as a probiotic microorganism in the food industry. However, further research and refinement of existing methods are needed to achieve a more accurate and reliable risk assessment.

### 3.8. Analysis of CRISPR/Cas Systems

Clustered, regularly interspersed, short palindromic repeats in the genome were identified using the online tool CRISPRfinder (Table 5) [16].

Currently, one of the universal defense mechanisms in the bacterial cell is considered to be CRISPR/Cas, which is a unique immune system [133,134].

Six genes for CRISPR-associated (cas) proteins belonging to class I (Cas3_0_I, Cas3_0_I, and Cas2_0_I-II-III), and class II Cas proteins (Cas9_0_II, Csn2_0_IIA, and Cas1_0_II) were identified in the genome of strain *L. paracasei* 2LB. During the analysis of the CRISPR-Cas system, two CRISPR loci were identified in the contigs “NODE_20” and “NODE_27” with Evidence Level 1 and 4, respectively.

The NODE_27 locus contains 31 unique spacers and proteins cas2_TypeI-II-III, cas1_TypeII, cas9_TypeII, and csn2_TypeIIA, which indicates a functionally complete type II CRISPR system (with Cas9 and Csn2), the presence of the strain’s immune memory, and long-term interaction with various phages or plasmids.

The presence of two unique spacers in the NODE_20 locus may indicate recent or incomplete adaptation to phage sequences. Therefore, it can be assumed that the CRISPR/Cas defense system is also involved in the evolution of bacteria by preserving information about previously encountered phages [135] and other mobile genetic elements [136].

It should be noted that improving the methodology for more accurate and reliable probiotic risk assessment and creating a single platform with an expanded set of genomic markers, combined algorithms, and in vitro/in vivo validation is an important and promising direction. The introduction of such an approach in future studies will improve the accuracy of assessment and reduce the likelihood of false positive and false negative results.

## 4. Conclusions

The objective of this in silico genome investigation was to identify genes that would allow for the assessment of the *L. paracasei* 2LB strain’s safety and probiotic properties, contributing to a better understanding of its applications. This is the first published complete genome sequence of *L. paracasei* isolated from Kazakh koumiss, with identification of unique gene clusters that may contribute to its adaptive characteristics. The strain is of particular interest due to in silico identification of genes responsible for probiotic characteristics such as resistance to osmotic stress, temperature extremes, acids, bile salts, and adhesion, as well as carbohydrate metabolism and bacteriocin production. The genome’s stability is further supported by the presence of two CRISPR loci, identified as protective mechanisms. Safety assessments revealed that strain 2LB is free of antibiotic resistance genes, virulence genes, and plasmid contigs.

Overall, this genomic analysis of key probiotic features and safety criteria indicates that strain 2LB could be a viable candidate for probiotics and starter cultures in functional food production. However, further research into its genomic and functional validation, along with in vitro and in vivo safety studies, is necessary for its successful application in the food industry and biomedicine.

## Figures and Tables

**Table 1:** Characteristics of *L. paracasei* 2LB: sequencing assembly and structural annotation.

Sequencing Assembly		Structural Annotation	
Assembled genome size, bp	3,066,038	G+C content (%)	46.25%
Number of contigs	219	CDS	2932
Longest contig, bp	195,833	tRNAs	59
Shortest contig, bp	129	tmRNAs	1
Contig number (>1000 bp)	75	rRNAs	6
Contig number (>5000 bp)	49	CRISPR number	2
Contig number (>10,000 bp)	40	Prophages	3 intact and 1 incomplete
Contig number (>25,000 bp)	32		
Contig number (>50,000 bp)	22		
Metric N50	105,408		
Metric N90	27,962		

**Table 2:** Analysis of carbohydrate-active enzymes (CAZy) in *L. paracasei* 2LB using the dCAZ3 server (85 genes). CAZy families: AA—auxiliary activities; CBM—carbohydrate-binding modules; CE—carbohydrate esterases; GH—glycoside hydrolases; GT—glycosyltransferases; PL—polysaccharide lyases.

CAZy Property	Numbers of Genes	Percentage (%)
AA	3	3.53
CBM	2	2.35
CE	5	5.88
GH	40	47.06
GT	33	38.82
PL	2	2.35
Total	85	

**Table 3:** Probiotic-related genes in the *L. paracasei* 2LB isolate.

Category	Role/Function	Genes	References
**Cold shock**	Cold shock protein	cspA	[59,60]
	Cold shock protein	cspB	[61]
	Cold shock protein	cspC	[62]
	Cold shock protein	cspG	[63,64]
	Cold shock protein	cspR	[65,66]
**Heat shock**	Chaperone protein	DnaK	[67,68]
	Heat shock protein	GrpE	[69]
	tmRNA-binding protein	SmpB	[70]
	DNA replication intiation control protein	YabA	[71,72]
	Translation elongation factor	LepA	[73,74]
	Chaperone protein	DnaJ	[75]
	Belongs to the small heat shock protein (HSP20) family	hsp1	[76]
	Belongs to the small heat shock protein (HSP20) family	hsp3	[76,77]
	Part of a stress-induced multi-chaperone system, it is involved in the recovery of the cell from heat-induced damage, in cooperation with DnaK, DnaJ and GrpE	clpC	[78,79]
	It cleaves misfolded, damaged, or unnecessary proteins and also plays a role in regulating cellular processes.	Clp protease	[80]
	serine protease	htrA	[80,81]
	Acts as a processive, ATP-dependent zinc metallopeptidase for both cytoplasmic and membrane proteins. Plays a role in the quality control of integral membrane proteins	ftsH	[82]
**Osmotic stress**	Glycine betaine ABC transport system, ATP-binding protein	OpuAA (EC 3.6.3.32)	[83]
	Glycine betaine ABC transport system, glycine betaine-binding protein	OpuAC	[84]
	Osmotically activated L-carnitine/choline ABC transporter, ATP-binding protein	OpuCA	[85]
	Osmotically activated L-carnitine/choline ABC transporter, permease protein	OpuCB	[86]
	L-proline glycine betaine ABC transport system permease protein	ProV (TC 3.A.1.12.1)	[87]
	Osmotically activated L-carnitine/choline ABC transporter, substrate-binding protein	OpuCC	[88]
	Glycine betaine ABC transport system, permease protein	OpuAB	[89]
	Osmotically activated L-carnitine/choline ABC transporter, permease protein	OpuCD	[88]
	L-proline glycine betaine binding ABC transporter protein	ProX (TC 3.A.1.12.1)	[87]
	Channel that opens in response to stretch forces in the membrane lipid bilayer. May participate in the regulation of osmotic pressure changes within the cell	mscL	[84]
**Bile salt**	Linear amide C-N hydrolases, choloylglycine hydrolase family	-	[90]
	Linear amide C-N hydrolases, choloylglycine hydrolase family	-	[91]
	Sodium bile acid symporter family	mleP	[92]
	Catalyzes the addition of an amino acid to the nucleotide precursor UDP-N-acetylmuramoyl-L-alanyl-D-glutamate (UMAG) in the biosynthesis of bacterial cell-wall peptidoglycan	murE	[51]
	Malic enzyme	mleS	[92]
**Adhesion**	Biosynthesis protein	epsB	[93,94]
	Glycosyltransferase like family 2	epsG	[95]
	Fibronectin-binding protein	FbpA	[14,96]
	MucBP domain	inlJ	[97,98]
	CHAP domain	p40	[99,100]
	NlpC P60 family protein	p75	[99,101]
	Transketolase, C-terminal domain protein	pdhB	[102]
	hydrolase, family 65, central catalytic	mapA	[103]
**pH**	Produces ATP from ADP in the presence of a proton gradient across the membrane	atpC	[104,105,106]
	ATP synthase (with catalytic β subunits like AtpD in bacteria) produces ATP from ADP using energy from a proton gradient	atpD	[105,106,107]
	ATP synthase produces ATP from ADP using a proton gradient, with the γ subunit regulating enzyme activity and proton flow through CF_0_	atpG	[106,108]
	ATP synthase produces ATP from ADP using a proton gradient, with its alpha subunit serving a regulatory role	atpA	[105,106]
	F_1_F_0_ ATP synthase generates ATP from ADP using a proton/sodium gradient, coupling catalytic F_1_ rotation with F_0_ proton translocation	atpH	[105,106]
	Component of the F(0) channel; it forms part of the peripheral stalk, linking F(1) to F(0)	atpF	[105,106]
	F_1_F_0_ ATP synthase harnesses proton/sodium gradients to drive ATP synthesis via rotational coupling between F_1_ catalytic core and F_0_ proton channel	atpE	[105,106]
	atpB plays a direct role in the translocation of protons across the membrane	atpB	[105,106,109]

**Table 4:** Identification of pathogenic genes using PHI-base v4.17.

Protein ID	Gene Name	Identity	Coverage	e-Value	Function	Gene ID
S4E4Q5	WalR	83.40	99.57	1.24 × 10^−115^	Transcriptional regulatory protein	EPH95667
Q82ZX2	CspR	89.39	98.51	6.13 × 10^−37^	Cold shock protein	AAO82613

**Table 5:** Characterization of CRISPR cassettes in *L. paracasei* 2LB.

Sequence	Spacer/Gene	CRISPR_Id	CRISPR_Start	CRISPR_End	CRISPR_Length	Direction	Consensus_Repeat	Evidence Level
NODE_20_length_63718_cov_19.819975	2	NODE_20_length_63718_cov_19.819975_crispr_1	41,374	41,641	267	ND	TATGTGGAGGTTTCTGCGACTGTGAGCGCGTTTCCGAGCGAAGCGTGGC	1
NODE_27_length_38928_cov_17.618979	31	NODE_27_length_38928_cov_17.618979_1	30,678	32,759	2081	ND	GTCTCAGGTAGATGTCGAATCAATCAGTTCAAGAGC	4

## Data Availability

This whole-genome sequencing project has been registered in DDBJ/ENA/GenBank under the number JBQPXO000000000 and under the BioProject identifier PRJNA1308329. The version described in this article is version JBQPXO010000000.

## Supplementary Materials

The following supporting information can be downloaded at: https://www.mdpi.com/article/10.3390/foods14193449/s1, Figure S1: In vitro pH and osmotic stress tolerance of *Lacticaseibacillus paracasei* subsp. *paracasei* 2LB strain; Table S1: Total of 93 whole-genome strains of *Lacticaseibacillus paracasei* taken from the NCBI database; Table S2: Analysis of carbohydrate-active enzymes (CAZymes) in the strain *Lacticaseibacillus paracasei* subsp. *paracasei* 2LB; Table S3: In silico safety and stability assessment. Table S4: In vitro evaluation of antagonistic activity, bile salt resistance, and adhesive properties (co-aggregation and auto-aggregation) of *Lacticaseibacillus paracasei* subsp. *paracasei* 2LB. References [137,138] are cited in the Supplementary Materials.
