# Supplementary material for: Lacticaseibacillus paracasei subsp. paracasei 2LB: Identification of Genes to Assess the Safety and Probiotic Potential of the Strain
Source: Foods. 2025 Oct 9;14(19):3449. doi: 10.3390/foods14193449 (PMC12523646; doi:10.3390/foods14193449)
Supplement: Supplementary file 1 [file foods-14-03449-s001.zip › Table S1. 93 whole genome strains of Lacticaseibacillus paracasei taken from the NCBI database.pdf]

Table S1. Identified *L. paracasei* strains with available complete genomes (n=93)

№	GenBank Accession (GCF ID)	Strain designation
1.	GCF_022701315.1	*L. paracasei* WX322
2.	GCF_003966835.1	*L. paracasei* IJH-SONE68
3.	GCF_013307125.1	*L. paracasei* Lp02
4.	GCF_025757505.1	*L. paracasei* NG-LCU-ST2
5.	GCF_026013725.1	*L. paracasei* ATG-E1
6.	GCF_024637915.1	*L. paracasei* VHProbi F11
7.	GCF_012955485.1	*L. paracasei* 347-16
8.	GCF_009931715.1	*L. paracasei* CACC 566
9.	GCF_001244395.1	*L. paracasei* L9
10.	GCF_902386635.1	*L. paracasei* MGYG-HGUT-02388
11.	GCF_030480425.1	*L. paracasei* L14
12.	GCF_017638905.1	*L. paracasei* HL182
13.	GCF_015476195.1	*L. paracasei subsp. tolerans* MGB0761
14.	GCF_015476175.1	*L. paracasei* MGB0747
15.	GCF_029625355.1	*L. paracasei* VHProbi OF10
16.	GCF_007637635.1	*L. paracasei* NJ
17.	GCF_025215665.1	*L. paracasei* ALAC-4
18.	GCF_025252385.1	*L. paracasei* VHProbi O44
19.	GCF_002902825.1	*L. paracasei* HDS-01
20.	GCF_002865565.1	*L. paracasei* HD1.7
21.	GCF_025917475.1	*L. paracasei subsp. paracasei* OLXI-3
22.	GCF_028609725.1	*L. paracasei* CLP-C10
23.	GCF_002079285.1	*L. paracasei* IIA
24.	GCF_008329845.1	*L. paracasei* 10266
25.	GCF_015693945.1	*L. paracasei subsp. tolerans* ZY-1
26.	GCF_004141835.1	*L. paracasei* SRCM103299
27.	GCF_037414215.1	*L. paracasei* LPC100
28.	GCF_041117985.1	*L. paracasei* MYA5
29.	GCF_003268715.1	*L. paracasei* LC355
30.	GCF_035586595.1	*L. paracasei* HP-B1337
31.	GCF_024498315.1	*L. paracasei* SMN-LBK
32.	GCF_016757695.1	*L. paracasei* S-NB
33.	GCF_016757675.1	*L. paracasei subsp. tolerans* S-NA5
34.	GCF_029925445.1	*L. paracasei* PC-H1
35.	GCF_015476135.1	*L. paracasei subsp. tolerans* MGB0734
36.	GCF_019175405.1	*L. paracasei subsp. paracasei* GR0548
37.	GCF_002813615.1	*L. paracasei subsp. paracasei* TMW 1.1434
38.	GCF_009739485.1	*L. paracasei subsp. paracasei* IBB3423
39.	GCF_024717335.1	*L. paracasei* VHProbi M56
40.	GCF_035330705.1	*L. paracasei* S135-24W
41.	GCF_002257625.1	*L. paracasei* TK1501
42.	GCF_003199005.1	*L. paracasei* Lpc10
43.	GCF_000155515.2	*L. paracasei subsp. paracasei* 8700:2

44.	GCF_003627255.1	*L. paracasei* ZFM54
45.	GCF_022819265.1	*L. paracasei* SCB0563
46.	GCF_000019245.4	*L. paracasei* Zhang
47.	GCF_018064185.1	*L. paracasei* HM1
48.	GCF_000418515.1	*L. paracasei* LOCK919
49.	GCF_040803205.1	*L. paracasei* B1
50.	GCF_025946725.1	*L. paracasei subsp. tolerans* FX-6
51.	GCF_025946745.1	*L. paracasei subsp. tolerans* FX-6-1
52.	GCF_027886355.1	*L. paracasei* NG-LCU-NJ2
53.	GCF_040536905.1	*L. paracasei* z15
54.	GCF_028609745.1	*L. paracasei* CLP-Y5
55.	GCF_030061895.1	*L. paracasei* GM-080
56.	GCF_000026485.1	*L. paracasei* BL23
57.	GCF_037414395.1	*L. paracasei* CUDS0725
58.	GCF_000194785.1	*L. paracasei* LC2W
59.	GCF_000194765.1	*L. paracasei* BD-II
60.	GCF_022478075.1	*L. paracasei* VHProbi F22
61.	GCF_024665815.1	*L. paracasei subsp. paracasei* 01
62.	GCF_008807095.1	*L. paracasei* TCS
63.	GCF_038098635.1	*L. paracasei subsp. tolerans* DS0725
64.	GCF_003957435.1	*L. paracasei* AO356
65.	GCF_007292115.1	*L. paracasei* CBA3611
66.	GCF_000388095.2	*L. paracasei* LcY
67.	GCF_037099855.1	*L. paracasei* DS2766
68.	GCF_015476075.1	*L. paracasei subsp. tolerans* MGB0245
69.	GCF_031348425.1	*L. paracasei* KCKM 0245
70.	GCF_021650835.1	*L. paracasei subsp. paracasei* GR0562
71.	GCF_040822445.1	*L. paracasei subsp. paracasei* BS2-PB5
72.	GCF_015476155.1	*L. paracasei subsp. tolerans* MGB0625
73.	GCF_025137495.1	*L. paracasei* L1
74.	GCF_014905075.1	*L. paracasei* NFFJ04
75.	GCF_023674545.1	*L. paracasei* 401
76.	GCF_015377585.1	*L. paracasei* TK-P4A
77.	GCF_034079445.1	*L. paracasei* A02
78.	GCF_964065205.1	*L. paracasei* CIRM-BIA2373
79.	GCF_022588775.1	*L. paracasei* BCRC-16100
80.	GCF_014905015.1	*L. paracasei* NSMJ15
81.	GCF_024158965.2	*L. paracasei* DM001
82.	GCF_001191565.1	*L. paracasei* CAUH35
83.	GCF_002442835.1	*L. paracasei* FAM18149
84.	GCF_009834405.1	*L. paracasei* TD 062
85.	GCF_018286435.1	*L. paracasei subsp. tolerans* 2A
86.	GCF_000014525.1	*L. paracasei* ATCC 334
87.	GCF_ 003177075.1	*L. paracasei* EG9
88.	GCF_040267725.1	*L. paracasei* TCI727
89.	GCF_030581835.1	*L. paracasei* XJ-003
90.	GCF_000582665.1	*L. paracasei* N1115

91.	GCF_001514415.1	*L. paracasei* KL1
92.	GCF_032466015.1	*L. paracasei* LMG 19719
93.	GCF_000829035.1	*L. paracasei subsp. paracasei* JCM 8130
