# Supplementary material for: Lacticaseibacillus paracasei subsp. paracasei 2LB: Identification of Genes to Assess the Safety and Probiotic Potential of the Strain
Source: Foods. 2025 Oct 9;14(19):3449. doi: 10.3390/foods14193449 (PMC12523646; doi:10.3390/foods14193449)
Supplement: Supplementary file 1 [file foods-14-03449-s001.zip › Table S4. In vitro evaluation of antagonistic activity, bile salt resistance, and adhesive properties (co-aggregation and auto-aggregation) of Lacticaseibacillus paracasei sub.pdf]

Table S4. *In vitro* evaluation of antagonistic activity, bile salt resistance, and adhesive properties (co-aggregation and auto-aggregation) of *Lacticaseibacillus paracasei* subsp. *paracasei* 2LB

In vitro tests showed that *Lacticaseibacillus paracasei* subsp. *paracasei* 2LB demonstrated notable antagonistic activity against target microorganisms and maintained growth in the presence of bile salts. The strain also exhibited pronounced adhesive capabilities, including both co-aggregation with pathogens and auto-aggregation, indicating strong colonization potential.

Table S4a. *In vitro* evaluation of antagonistic activity of *Lacticaseibacillus paracasei* subsp. *paracasei* 2LB

Strain name	Antagonistic activity (mm)			
	Salmonella typhimurium	*Staphylococcus aureus*	*Candida albicans*	*Escherichia coli*
Lacticaseibacillus paracasei subsp. *paracasei 2LB*	30,43±1,40	17,27±0,51	17,70±0,75	23,60±1,40

Note: P<0.05

Inhibition zones: 0 mm – zero activity; 1.0–4.9 mm – low activity; 5.0–8.9 mm – medium; ≥ 9 mm – high

The antagonist activity of the isolated probiotic strain 2LB was assessed using the agar well diffusion assay. To evaluate the inhibitory potential of the probiotic, indicator microorganisms such as *Escherichia coli* ATCC 25922 B-RKM 0447, *Salmonella typhimurium* TA 98 B-RKM 0162, *Staphylococcus aureus* 209 P B-RKM 0057, and *Candida albicans* ATCC 885-653 Y-RKM 0475 were used. The surface of the agar nutrient medium in Petri dishes was evenly seeded with indicator strain cultures to form a bacterial lawn. Wells (10 mm in diameter) were created in the agar and filled with 100 µl of the overnight probiotic strain culture. The wells were incubated for 30 minutes at room temperature to ensure uniform distribution. The plates were then incubated overnight at the optimal growth temperature for each indicator strain. After incubation, the plates were examined for clear zones of inhibition around the wells, indicating the antagonistic effect of the probiotic strain on the test microorganisms.

Reference: Abitaeva, G.K., et al. (2022). Characteristics of probiotic strains for the development of preventive drinks. Microbiology and Virology, 4(39), 142–158. https://doi.org/10.53729/MV-AS.2022.04.11

Table S4b. *In vitro* evaluation of bile salt resistance of *Lacticaseibacillus paracasei* subsp. *paracasei* 2LB

Conc. / Strain name	0,3% bile, CFU/mL	0.5% bile, CFU/mL	1% bile, CFU/mL
Lacticaseibacillus paracasei subsp. paracasei 2LB	$1,87\times10^{7}$	$3,90\times10^{6}$	$4,73\times10^{5}$

To assess bile resistance, strain 2LB were cultured in MRS broth using bile solutions prepared from Oxbile powder (HiMedia, India) dissolved in MRS broth to achieve final concentrations of 0.3%, 0.5%, and 1%. One milliliter of fresh overnight bacterial culture was centrifuged at 7000 rpm for 5 minutes, after which the pellet was washed twice with 0.9% saline. The resulting pellet was then mixed with MRS broth containing the appropriate bile concentrations at a ratio of 1:10. After incubation at 37°C for 12–13 hours, the number of viable cells was

determined by serial dilution, followed by plating on MRS agar plates and counting the colony-forming units (CFU).

Reference: Talib et al. Isolation and Characterization of Lactobacillus spp. from Kefir Samples in Malaysia // Molecules. 2019. Vol. 24, № 14. P. 2606. https://doi.org/10.3390/molecules24142606

Table S4c. *In vitro* evaluation adhesive properties (coaggregation and auto-aggregation) of *Lacticaseibacillus paracasei* subsp. *paracasei* 2LB

Autoaggregation (%)	Coaggregation rates (%)			
	Escherichia coli	Candida albicans	Salmonella typhimurium	Staphylococcus aureus
83,85	14,51	6,79	15,46	15,46

The autoaggregation ability of 2LB was evaluated using the method described by Kos et al., with minor modifications. The 2LB culture, grown overnight at 37 °C, was harvested by centrifugation at 7000 × g for 20 min and washed twice with phosphate-buffered saline (PBS). The resulting cell suspensions were vortexed for 1 min. Autoaggregation was determined after 3 h and 5 h of incubation at room temperature. The upper suspension (0.2 mL) was mixed with 0.8 mL PBS, and the optical density (OD) was measured immediately. The percentage of autoaggregation was calculated as: $A = 1 - \left({A_{t5}}/{A_{t0}}\right) * 100\%$, where A_{t5} is the absorbance measured at 600 nm after 5 h (using a microplate reader, Biochrom) and A_{t0} - is the absorbance at time zero.

Coaggregation was assessed using the same procedure as for autoaggregation. Fresh 2LB suspension was diluted to $1{\times}10^8$ CFU/mL in PBS. Equal volumes (2 mL each) of the suspension were mixed with suspensions of pathogenic strains (*Escherichia coli* ATCC 25922 B-RKM 0447, *Salmonella typhimurium* TA 98 B-RKM 0162, *Staphylococcus aureus* 209 P B-RKM 0057, and *Candida albicans* ATCC 885-653 Y-RKM 0475). The mixtures were gently mixed and left to settle at room temperature. Control tubes containing 4 mL of each bacterial suspension alone were prepared. Absorbance was measured at 600 nm after 5 h of incubation at 37 °C. The percentage of coaggregation was calculated using the formula: $\%\ \text{coaggregation} = \frac{\frac{A_x + A_y}{2} - A(x+y)}{A_x + {A_y}/{2}}$, where *Ax* and *Ay* are the absorbance values of each strain in control tubes, and *A(x+y)* is the absorbance of the mixture.is the absorbance of the mixture.

Reference: Kos B., Susković J., Vuković S., Simpraga M., Frece J., Matošić S. (2003). Adhesion and aggregation ability of probiotic strain Lactobacillus acidophilus M92. Journal of Applied Microbiology, 94(6), 981–987. https://doi.org/10.1046/j.1365-2672.2003.01915.x
